# Robot-assisted thoracic surgery for stages IIB–IVA non-small cell lung cancer: retrospective study of feasibility and outcome

**DOI:** 10.1007/s11701-023-01549-3

**Published:** 2023-03-16

**Authors:** Ghada M. M. Shahin, Peter-Paul W. K. Vos, Merlijn Hutteman, Jos A. Stigt, Jerry Braun

**Affiliations:** 1 Department of Cardiothoracic Surgery, Leiden University Medical Center, Leiden, The Netherlands; 2 Department of Cardiothoracic Surgery, University Medical Center Utrecht, Utrecht, The Netherlands; 3 Department of Surgery, Leiden University Medical Center, Leiden, The Netherlands; 4 Department of Pulmonology, Isala, Zwolle, The Netherlands

**Keywords:** Robot-assisted thoracic surgery, Non-small lung carcinoma, Higher stages, Locally advanced, Video-assisted thoracic surgery

## Abstract

Robot-assisted thoracic surgery (RATS) for higher stages non-small cell lung carcinoma (NSCLC) remains controversial. This study reports the feasibility of RATS in patients with stages IIB–IVA NSCLC. A single-institute, retrospective study was conducted with patients undergoing RATS for stages IIB–IVA NSCLC, from January 2015 until January 2020. Unforeseen N2 disease was excluded. Data were collected from the Dutch Lung Cancer Audit database. Conversion rate, radical (R0) resection rate, local recurrence rate and complications were analyzed, as were risk factors for conversion. RATS was performed in 95 patients with NSCLC clinical or pathological stages IIB (*N* = 51), IIIA (*N* = 39), IIIB (*N* = 2) and IVA (*N* = 3). 10.5% had received neoadjuvant chemoradiotherapy. Pathological staging was T3 in 33.7% and T4 in 34.7%. RATS was completed in 77.9% with a radical resection rate of 94.8%. Lobectomy was performed in 67.4% of the total resections. Conversion was for strategic (18.9%) and emergency (3.2%) reasons. Pneumonectomy (*p* = 0.001), squamous cell carcinoma (*p* < 0.001), additional resection of adjacent structures (p = 0.025) and neoadjuvant chemoradiation (*p* = 0.017) were independent risk factors for conversion. Major post-operative complications occurred in ten patients (10.5%) including an in-hospital mortality of 2.1% (*n* = 2). Median recurrence-free survival was estimated at 39.4 months (CI 16.4–62.5). Two- and 5-year recurrence-free survival rates were 53.8% and 36.7%, respectively. This study concludes that RATS is safe and feasible in higher staged NSCLC tumors after exclusion of unforeseen N2 disease. It brings new perspective on the potential of RATS in higher stages, dealing with larger and more invasive tumors.

## Introduction

For early-stage non-small cell lung carcinoma (NSCLC), surgical resection is the treatment of choice. To minimize the effects of surgery on patient recovery, minimally invasive surgery (MIS) is now widely implemented. MIS entails multiportal and uniportal video-assisted thoracoscopic surgery (VATS), as well as robot-assisted thoracic surgery (RATS) [1] [2]_._ MIS is associated with faster recovery after surgery, less complications and better post-operative quality of life compared to open thoracotomy [3–8] Therefore, MIS has become the treatment of choice in early-stage NSCLC and its application is being studied in locally advanced disease (TNM stage III) [9].

Ever since its clinical introduction in 2002, RATS is increasingly used as it offers technical advantages over VATS, potentially allowing surgeons to radically resect more advanced tumors in a minimally invasive way. Studies comparing RATS with VATS in early-stage NSCLC demonstrate its potential benefit in terms of less complications, less blood transfusions, increased lymph node harvesting and even improved oncological efficacy compared to VATS [8, 10–12]. In addition, RATS shows lower rates of conversion to open thoracotomy. [13, 13]. A randomized clinical trial [15] in 320 patients with early-stage NSCLC found comparable peri-operative outcomes between VATS and RATS, with RATS having a significantly higher number of harvested lymph nodes.

Literature on RATS in locally advanced stages of NSCLC often involves patients with lymph node involvement (N2 disease). These studies emphasize that the potential of RATS is to yield more lymph nodes, which might improve survival [3, 11, 16, 17]. Nevertheless, the application of MIS in locally advanced lung cancer as defined by higher T stages (TNM T3-T4) remains a matter of debate. Studies examining RATS in this subgroup of patients are scarce, with relatively small sample sizes [18–20]. These often larger-sized, more centrally located tumors are associated with bronchial and vascular invasion, making a radical resection a greater challenge. Robotic surgical platforms can possibly facilitate these resections, as they offer a series of technological features that are unavailable with VATS, resulting in increased access for minimal invasive surgical treatment.

The aim of this study is to analyze the feasibility and outcomes of RATS in stages IIB–IVA NSCLC tumors (8th edition TNM [21]. It resulted in the evaluation of a group of patients with larger-sized lung tumors, without shying away from nodal involvement, neoadjuvant chemoradiotherapy or involvement of adjacent structures. It was decided not to include patients in stage IIA (non-invasive tumors > 4 cm and ≤ 5 cm, N0), as they do not often pose a surgical challenge. Patients with unforeseen N2 disease were excluded and IVA was included as it concerned oligometastatic disease. Conversion rate (including reasons for conversion and predictors), completeness of resection (R0 resection rate), post-operative complications and recurrence-free survival were analyzed.

## Methods

This is a single-institute, single-console surgeon, retrospective study. In the Isala hospital, Zwolle, the Netherlands, the RATS program was started in October 2011. At that time, the minimally invasive surgical technique of choice was VATS. By 2013, all minimally invasive oncologic lung resections were performed by RATS, leaving VATS for the treatment of benign diseases, such as pneumothorax, empyema and diagnostic wedge resections. Upfront open thoracotomy was reserved for patients who were not suitable for MIS (e.g., patients in need of emergency salvage surgery). In this study, the results of complex lung resections are described as an illustration of the feasibility of RATS, without trying to make a comparison with other approaches such as thoracotomy or VATS. The current study was written according to STROBE guidelines [22].

### Selection of patients

This study includes consecutive patients with NSCLC who underwent RATS from January 2015 till January 2020. We did not perform any selection of the patients for the current study other than the clinical, pathological and post-chemoradiotherapy TNM stage. There was one exclusion criterion concerning patients with unforeseen N2 disease. All patients were in either clinical (cTNM), pathological (pTNM) or after neoadjuvant therapy (ypTNM) stage IIB–IVA. Patients in clinical stage IVA were diagnosed with oligometastatic disease and included intentionally with curative intent, and the metastases did not interfere with the resectability and operability of the primary lung cancer. Moreover, they would have been staged as stage II–III if no oligometastases had been diagnosed. Patients with carcinoid tumors were included in the general analysis. However, they were excluded from survival and recurrence analyses because of their different (typically more favorable) tumor biology. Patients with clinical N2 disease and T4 tumors received neoadjuvant chemoradiotherapy prior to surgical resection and were included in the study as they represent the technically most challenging operations.

### Data collection

Patient data were obtained from the Dutch Institute of Clinical Auditing (Dutch Lung Cancer Audit “DLCAS-2019 Version 1.0.3”). Ethical review and approval were waived for this study, as all patients agreed upon admission to register their data in the national database (DLCA). General patient characteristics were collected, including American Society of Anesthesiologists (ASA) and performance status according to Eastern Cooperative Oncology Group (ECOG).

### Staging

Pre-operative mediastinal staging was conducted with positron emission tomography–computed tomography (PET-CT) according to international guidelines. When indicated, patients subsequently underwent either endo-esophageal ultrasound (EUS) and/or endobronchial ultrasound (EBUS) or mediastinoscopy. Tumor stage and invasion were determined according to the 8th International Association for the Study of Lung Cancer Tumor, Node, Metastasis, Staging System (TNM). We defined pre-operative vascular invasions as invasion of the central vessels, such as left or right main pulmonary artery or left or right pulmonary veins [21]. All patient data were discussed by a multidisciplinary team consisting of oncologic pulmonologists, a radiologist, a radiotherapist and a thoracic surgeon.

### Learning curve

The console surgeon started to use RATS for lung resections in November 2011. By 2013, the learning curve had progressed in a way that all oncologic lung resections were performed robotically, and VATS was only used for benign diseases. By the start of this study in January 2015, 240 RATS cases had been performed with a dedicated team comprising one anesthetist, two anesthesia technicians, one bedside assistant and four scrub nurses. The learning curve for surgery of more advanced disease is an ongoing, maybe never-ending, process. Patient selection is key, when a team is starting a new surgical technique and small peripheral tumors with prior induction therapy are the ones to start with [23]. In patients with advanced NSCLC, surgery was started as RATS and converted strategically when considered necessary.

### Surgical technique

The Intuitive Surgical da Vinci Si platform (Intuitive Surgical Inc., Sunnyvale CA, USA) was used in all cases. Technologic features, such as Endowrist^TM^ technology, scaling down of motion, length of the instruments, three-dimensional high-definition camera, the ability to use three instruments simultaneously and tremor filtration were all accessible on the device. All patients were operated with a totally endoscopic four-port surgical approach, using CO_2_ insufflation and an assistant port. The ports were placed in the fifth or sixth intercostal space with a 9 cm distance to each other, preferably in a similar intercostal space. The assistant port was placed in the eighth or ninth intercostal space in a triangle configuration between the ports for arms 1 and 2. The assistant port was enlarged to a maximum length of 8 cm to extract the specimen at the end of the operation. The ribs were neither spread nor cut. With increasing experience, we found it preferable to extract large parenchyma-infiltrating tumors by enlarging the camera port, as this causes less pain and rib dislocation.

### Lymph node dissection

Lymph node dissection is not only one of the major components of lung cancer surgery, but is also key to facilitate lung resection. We, therefore standardized our technique in a way that the lymph node dissection was carried out first. Right upper lobe stations are removed in the following order: 2, 4, 10, 7 and 11. Left upper lobe stations are removed in the order of 5, 6, 7, 10 and 11, and the lower lobes stations in the order 9, 8, 7, 10 and 11. The technical features of the robot platform are valuable, especially in challenging lymph nodes, such as the ones that are adherent to arteries or the subcarinal nodes in left sided cases. The value of RATS in lymph node dissection is further elaborated in our recently published article [24].

### Peri-operative data

Peri-operative data collected were tumor histology and location, intra-operative blood loss, conversion to thoracotomy and reasons for conversion. Post-operative data included complication rates (classified according to Clavien–Dindo [25]), local recurrence rate, occurrence of distant metastases and median (recurrence-free) survival. Post-operative complications were assessed up to 30 days. Follow-up data were gathered by reviewing the patient’s hospital files until the last available date.

Pathologic assessment included size, type of tumor and radicality of resection. To identify independent risk factors associated with conversion to thoracotomy, surgical characteristics and conversions were analyzed for the reasons to convert and compared to the “completion of RATS group’’. Follow-up was performed by the pulmonologist with repetitive CT scans. Since evidence-based recommendations for timing and frequency of follow-up imaging were only recently published in the ASCO guidelines [26], patients in our database received no CT scans at standardized intervals per protocol. Nevertheless, scans were typically made on a semi-annual or annual base. In case of missing data, patients or their relatives were contacted for completion of survival data. If not available, patients were excluded.

### Statistical methods

SPSS statistics version 28.0 was used for statistical analysis. Normally distributed data were expressed as mean and standard deviation (± SD). Non-normally distributed data were expressed as median and interquartile range (IQR). Chi square and Fisher exact tests were used for analyzing differences between the groups.

In this retrospective observational study, we tried to adjust for potential bias. A binary logistic regression analysis was performed to compare group differences between conversion and non-conversion groups, retaining variables with *p* < 0.05 in the final multivariable model. Variables proved significant in univariate analysis (*p* < 0.05) were used for multivariate analysis and entered in a multivariable model in forward regression algorithm.

Median recurrence-free survival (months) was assessed using Kaplan–Meier plotting during which right censoring was applied Kaplan–Meier survival analysis was performed to estimate mean survival of total group. Separate group analysis was performed for mean survival of patients who had either no local recurrence, or local recurrence, or local recurrence plus distant metastasis or distant metastasis only.

## Results

### Baseline and surgical characteristics

This cohort consisted of 95 patients with a median age of 72 years (64–77), of which 67.4% were male. (Table 1). Patients were pre-operatively scored as ASA 1 in 44.2% and 81.1% of the patients had a normal ECOG score. An average percentage of forced expiratory volume per second (FEV1) was predicted at 75% (± 16%) and total lung capacity at 83% (± 18%). Data on lung function tests FEV1 and TLC and VO_2_max were reported when available (Table 1). The tumor showed tracer uptake on PET–CT in 96.8% of the patients. Invasive mediastinal staging was performed in 80.9% of patients: EBUS (57.9%), EUS (14.7%) or both (8.3%). Additional mediastinoscopy was performed in 2.1% of patients.

**Table 1:** Patient characteristics

Patient characteristics	*n* = 95	Surgical resection	
Age median IQR (years)	72 (64–77)	RUL	29 (30.5%)
Male	64 (67.4%)	RML	5 (5.3%)
BMI	26 (± 4.4)	RLL	19 (20.0%)
		LUL	26 (27.4%)
ASA score		LLL	17 (16.8%)
1	42 (44.2%)		
2	30 (31.6%)	Surgical approach	
3	21 (22.1%)	Lobectomy	64 (67.4%)
4	2 (2.1%)	Bilobectomy	9 (9.5%)
		Segmentectomy	1 (1.1%)
ECOG		Sleeve	4 (4.2%)
Normal	77 (81.1%)	Pneumonectomy	17 (17.9%)
1	14 (14.6%)		
2	4 (4.2%)	Invasion total	
		Total of patients	44 (46.3%)
Lung function predicted %		Pleural	26 (27.4%)
Total lung capacity	83 (± 18)	Thoracic wall	6 (6.3%)
FEV1	75 (± 16)	Mediastinal	6 (6.3%)
		Vascular	12 (12.6%)
Tumor staging		Bronchial	2 (2.1%)
PET positive	93 (96.8%)	Phrenic nerve	1 (1.1%)
EBUS	55 (57.9%)	Pericardial	3 (3.2%)
EUS	14 (14.7%)		
EBUS and EUS	8 (8.3%)	Radical resection rate	
Mediastinoscopy	2 (2.1%)	R0	90 (94.8%)
Clinical N2 disease	9 (9.5%)	R1	4 (4.2%)
Clinical N1 disease	14 (14.7%)	R2	1 (1.1%)
		Type of conversion	
TNM (8^th^ edition)		RATS completion	71 (74.7%)
IIB	51 (53.7%)	Mini-thoracotomy	1 (1.1%)
IIIA	39 (41.0%)	Sternotomy for CABG	2 (2.1%)
IIIB	2 (2.1%)	Conversion thoracotomy	21 (22.1%)
IVA	3 (3.2%)	Elective	18 (18.8%)
		Emergency	3 (3.2%)
Histology			
Adenocarcinoma	48 (50.5%)	Conversion reason	
Squamous cell carcinoma	42 (44.2%)	Elective	18 (18.8%)
Miscellaneous carcinoids	4 (4.3%)	Chest wall resection	2 (2.1%)
Large cell carcinoma	1 (1.1%)	Pericardial resection	2 (2.1%)
Lymph nodes dissected	6.1 (± 1.5)	Vascular invasion	6 (6.3%)
		Bulkiness of tumor	4 (4.2%)
Induction therapies	10 (10.5%)	No robotic port entry	1 (1.1%)
Conc. chemoradiation	1 (1.1%)	Tear pars membranous	2 (2.1%)
Seq. chemoradiation	7 (7.4%)	Development fissure	1 (1.1%)
Chemotherapy	1 (1.1%)	Emergency	3 (3.2%)
Radiotherapy	1 (1.1%)	Bleeding	3 (3.2%)
No	85 (89.5%)		
		Recurrence (NSCLC n = 91)	
Tumor characteristics		Local recurrence	7 (7.7%)
Tumor diameter mm	55 (35–70)	Metastasis	15 (16.5%)
Centrally located	21 (22.1%)	Both	15 (16.5%)
		Local recurrence rate	22 (24.2%)

Fifty-one patients (53.7%) were in stage IIB, 39 (41.0%) in stage IIIA, 2 (2.1%) in stage IIIB and 3 (3.2%) in stage IVA. Histology showed adenocarcinoma in 50.5% of the patients, squamous cell carcinoma in 44.2%, large cell tumor in 1.1% and carcinoid tumors in 4.2%.

Ten patients (10.5%) received induction therapy prior to surgery, mostly sequential chemoradiotherapy. The indications for neoadjuvant therapy were N2 lymph nodes status or large tumor size. In 22.1% of the patients, the tumor was located centrally. Median tumor diameter was 55 mm (34–70), with the right upper lobe being the most common location.

Most patients underwent a lobectomy (67.4%, *N* = 64), followed by pneumonectomy in 17.9% (*N* = 17), bilobectomy (9.5%, *N* = 9), bronchial sleeve lobectomy (4.2%, *N* = 4) and segmentectomy (1.1%, *N* = 1).

Histologic examination showed 44 patients (46.3%) to have invasion in adjacent structures, either at one or multiple locations. Sites of invasion were parietal pleura (*n* = 26), thoracic wall (*N* = 6), mediastinum (*N* = 6), vascular (*N* = 12), bronchial (*N* = 2), phrenic nerve (*N* = 1) or pericardium (*N* = 3). Microscopically radical resection was achieved in 94.8% of patients. Of the five patients in whom a radical resection was not achieved, four were operated in the first 2 years of the study and were scored as local recurrence. Intra-operative blood loss was < 500 ml in 82.5% of the patients. Overall, pathological tumor (pT) stage showed a majority of T3 (*N* = 32) and T4 (*N* = 33) tumors. In the current study, 6.1 (± 1.5) lymph node stations were dissected.

Clinical N1 lymph node status was 14.7%, but pathologic N1 status was present in 38.9%. Clinical N2 disease was present in 9.5% (*N* = 9), whereas pathological examination showed N2 disease in 5.3%. This downstaging can be explained from the fact that these nine patients received neoadjuvant chemoradiation. A discrimination between single level and multi-level N2 disease was not made, although an argument can be made to offer upfront surgery to patients with single level N2 disease followed by adjuvant therapy.

Three patients had oligometastatic disease. These metastases were treated after resection of the primary lung carcinoma. The metastases had no effect on the resectability of the lung tumor or the operability of the patients.

### Conversion to thoracotomy

The resection was completed via RATS in 74 patients (77.9%), whereas a conversion to thoracotomy was performed in 21 cases (22.1%). Eleven (17.2%) lobectomies, 9 (52.9%) pneumonectomies and 1 (11.1%) bilobectomy were converted to thoracotomy. Median tumor diameter in the pneumonectomy group was 65 mm (46–80). In total, 21 conversions were performed, of which 3 were in emergency setting due to intra-operative bleeding. In the remaining 18 patients, conversion was chosen for strategic considerations. These considerations were to achieve radical resection in case of arterial, pericardial or chest wall invasion (*n* = 10) or bulkiness of the tumor (*n* = 4). Other reasons were dense pleural adhesions hindering placement of operating ports (*n* = 1), absent interlobar fissure (*n* = 1) and a tear in the membranous part of main the bronchus (*n* = 2); in both latter cases, neoadjuvant chemoradiation had been administered prior to surgery (Table 1). In seven of these converted patients the tumor was located centrally.

Additional surgery was performed in eight patients. Three patients received en bloc chest wall resection and three patients en bloc pericardial resection. Two patients had concomitant coronary artery bypass grafting through median sternotomy after completion of the RATS procedure. In one patient, the tumor size justified a 10 cm mini-thoracotomy for removal of the resected lobe. The latter three patients were not considered to be conversions.

### Independent risk factors of conversion

Baseline and surgical characteristics were analyzed comparing patients who had surgery completed by RATS or in whom conversion to thoracotomy was performed (Table 2). The conversion group showed significantly higher rates of central tumor location pneumonectomy, general tumor invasion, vascular invasion, left upper lobe resection, squamous cell histology, induction therapy and additional resection of adjacent structures such as pericardium or chest wall.

**Table 2:** Group differences associated with conversion in both groups

Predictors of conversion	No conversion *n* = 74	Conversion *n* = 21	*p* value (*p* < 0.05)
Age > 65	53 (70.6%)	13 (61.9%)	0.48
BMI > 25	39 (52.0%)	11 (52.4%)	0.98
ASA (IQR)	2 (1–3)	2 (1–2)	0.20
Tumor size > 50 mm	41 (54.6%)	9 (42.9%)	0.38
Central tumor	27 (36.4%)	14 (66.7%)	**0.046**
Pneumonectomy	8 (10.8%)	9 (42.9%)	** < 0.001**
Lobectomy	57 (77.0%)	11 (52.4%)	**0.025**
General invasion	29 (38.6)	15 (71.4%)	**0.009**
Pleural invasion	20 (26.7%)	6 (28.6%)	0.47
Vascular invasion	5 (6.7%)	7 (33.3%)	**0.001**
Left upper lobe	14 (18.7%)	12 (57.1%)	** < 0.001**
Squamous cell (histology)	25 (33.3%)	17 (81.0%)	** < 0.001**
Induction therapy	5 (6.8%)	5 (23.8%)	**0.025**
Additional Surgery	3 (4.1%)	5 (23.8%)	**0.004**

Statistically significant values (*p* < 0.05) in bold

Univariate analysis showed all but central tumor location (*p* = 0.49) and lobectomy (*p* = 0.29) to be significantly associated with conversion. Multivariate analyses of remaining significant variables (*p* < 0.05) showed pneumonectomy (odds ratio OR 19.4; CI 3.2– 119.6, *p* = 0.001), squamous cell histology (OR 20.3; CI 3.3–126.8, *p* < 0.001), induction therapy (OR 16.0; CI 1.6–156.5), *p* = 0.017) and additional resection of adjacent structures. (OR 15.2; CI 1.4–165.1, *p* = 0.025) to be independent risk factors for conversion to thoracotomy (Table 3).

**Table 3:** Binary logistic regression model for independent risk factors associated with conversion to thoracotomy at *p* < 0.05 included in the model

Predictors of conversion	Univariate (OR; CI 95%)	Multivariate (OR; CI 95%)
Pneumonectomy	*P* < 0.001 (6.8; 2.2–20.7)	*P* = 0.001 (OR 19.4; CI 3.2– 119.6)
Squamous cell (histology)	*P* < 0.001 (OR 6.5; CI 2.2–19.8)	*P* < 0.001 (OR 20.3; CI 3.3–126.8)
Induction therapy	*P* = 0.036 (OR 4.3; CI 1.1–16.5)	*P* = 0.017 (OR 16.0; CI 1.6–156.5)
Additional resection of adjacent structures	*P* = 0.007(OR 21.6; CI 2.4–197.5)	*P* = 0.025 (OR 15.2; CI 1.4–165.1)
General invasion	*P* = 0.017 (OR 3.4; CI 1.3–9.5)	*P* = 0.9 (OR 0.89; CI 0.15–5.2)
Vascular invasion	*P* = 0.005 (OR 6.4; CI 1.8–22.8)	*P* = 0.87 (OR 1.2; CI 0.18–7.6)
Left upper lobe	*P* < 0.001 (OR 6.7; CI 2.4–18.9)	*P* = 0.11 (OR 3.3; CI 0.8–14.4)
Central tumor	*P* = 0.16 (OR 1.2; CI 0.92–1.7)	Not significant univariate
Lobectomy	*P* = 0.29 (OR 0.6; CI 0.23–1.6)	Not significant univariate

### Post-operative complications

There were 44 post-operative complications in a total of 34 patients (35.8%) (Table 4). Thirty-two (72.7%) were grade I–II Clavien–Dindo complications, of which 19 were treated conservatively (grade I), including post-operative air leak (*n* = 17), recurrent nerve injury (*n* = 1) and radial nerve neuropathy (*n* = 1). Thirteen complications needed pharmacological (grade II) intervention for either infection, supraventricular tachycardia, or pulmonary edema.

**Table 4:** Complications according to Clavien–Dindo classification

Clavien–Dindo (34 patients)	Complications (44 total)	95 patients
Grade I Any deviation from the normal post-operative course without the need for pharmacological treatment or surgical, endoscopic and radiological interventions	(Total = 19)	
	Solitary air leak > 5 days	17 (17.9%)
	Radialis neuropathy	1 (1.1%)
	Recurrent laryngeal nerve injury	1. (1.1%)
Grade II Requiring pharmacological treatment with drugs other than that allowed for grade I complications	(Total = 13)	
	Pneumonia	5 (5.3%)
	Ileus	1 (1.1%)
	Urinary tract infection	1 (1.1%)
	Supra ventricular tachycardia	4 (4.2%)
	Lung edema	2 (2.1%)
Grade III Requiring surgical, endoscopic or radiological intervention	(Total = 1)	
	Empyema (alteplase)	1 (1.1%)
Grade IIIb Interventions under general anesthesia	(Total = 8)	
	Empyema (VATS)	1 (1.1%)
	Empyema (VATS)	1 (1.1%)
	Prolonged air leak (VATS)	4 (4.2%)
	Bronchopleural fistula	1 (1.1%)
	Haematothorax	1 (1.1%)
Grade IV Life-threatening complication including CNS complications requiring IC/ICU management	(Total = 1)	
	Cerebrovascular Accident	1 (1.1%)
Grade V Death of a patient	(Total = 2)	2 (2.1%)
	Cerebrovascular accident	
	In-hospital cardiac arrest	

Major complications, grade III–IV, occurred in ten patients (10.5%). Nine were re-interventions, one percutaneous alteplase treatment for empyema, eight patients underwent a re-intervention under general anesthesia, including VATS for persisting air leakage (*n* = 4) and empyema (*n* = 2). One patient needed emergency surgery for post-operative hemothorax, and one patient developed a bronchopleural fistula.

Post-operative mortality rate was 2.1% (grade V complication) due to myocardial infarction (*n* = 1) and cerebrovascular accident (*n* = 1), both complications could not be attributed to the use of the surgical robot.

### Survival and recurrence

**Fig. 1:**
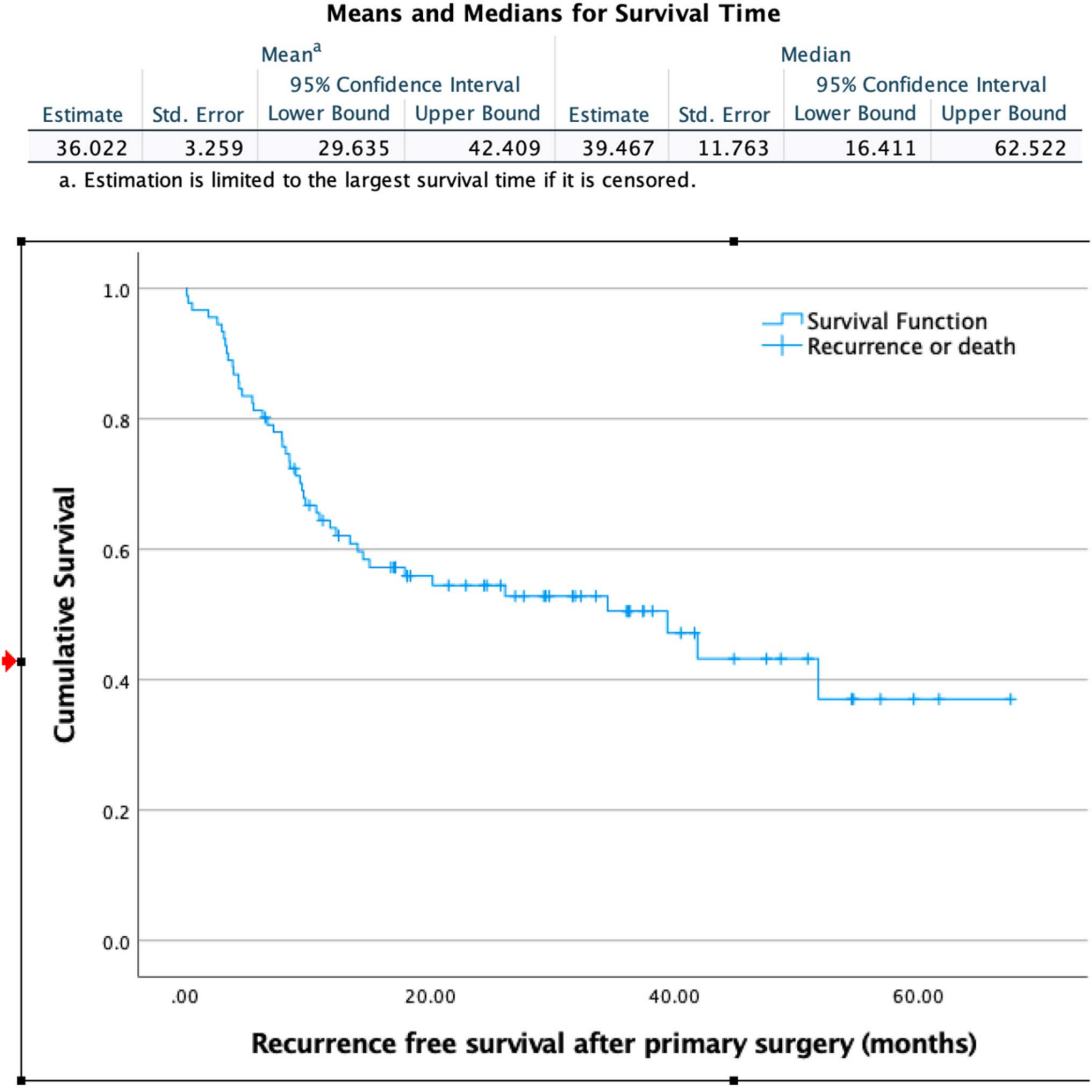
Recurrence-free survival in months. Carcinoid tumors are excluded

**Fig. 2:**
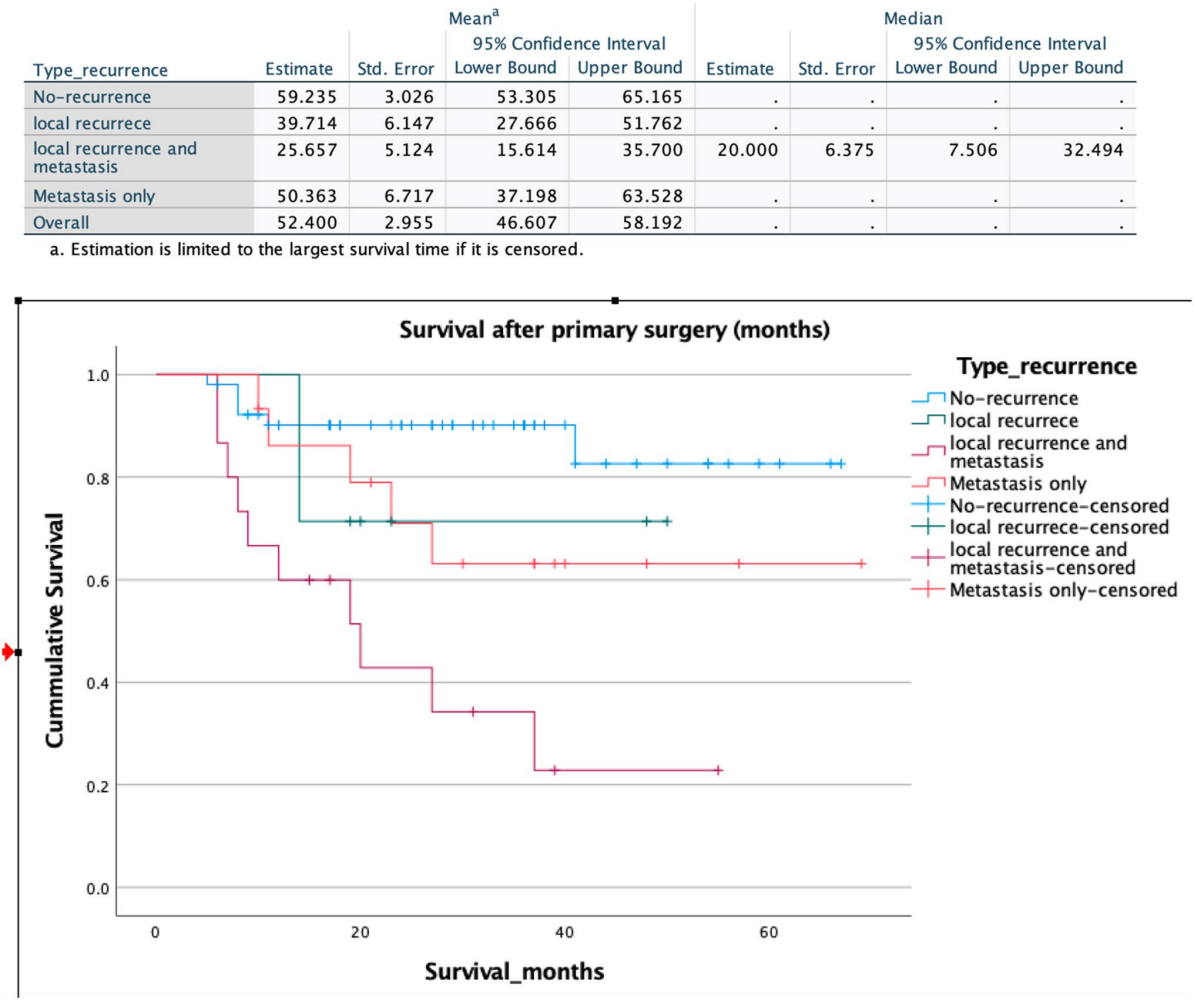
Kaplan–Meier survival curve. Estimated mean survival per type of recurrence

A total of 37 (41.5%) patients developed either local recurrence (*n* = 7), local recurrence combined with distant metastasis (n = 15) or distant metastasis alone (*n* = 15). This resulted in a total local recurrence rate of 24.7%. Median recurrence-free survival was 39.4 months (CI 16.4–62.5) (Fig. 1). Two- and 5-year recurrence-free survival rates were 53.8% and 36.7%, respectively. Kaplan–Meier survival analysis showed an overall mean estimated survival of 52 months (CI 46.6–58.1) Fig. 2 shows survival per type of recurrence with calculated mean survival accordingly.

## Discussion

This study analyzed the feasibility of RATS in higher-stages (IIB-IVA) NSCLC, characterized by larger tumor size, central tumor location and invasion of vital structures and some resections were performed after neoadjuvant therapy. The feasibility is expressed in terms of radicality of resection, rate of conversion to thoracotomy, local recurrence rate and complication rate.

### Radicality

The radicality rate that was reached in this study was 94.8% (R0: *N* = 90 (94.8%) R1: *N* = 4 (4.2%) R2: *N* = 1 (1.1%)).and is comparable with the radicality in reports of previous cohorts varying from 94.1% to 98.4%.

In a retrospective analysis by Li comparing RATS (*n* = 36) to VATS (*n* = 85) lobectomies in cTNM IIB–IIIA NSCLC, a radical resection rate of 94.4% was reported and was similar to VATS [17].

A multi-center retrospective study performed by Veronesi and co-workers, evaluating RATS in 210 patients with locally advanced NSCLC based on clinically evident or occult N2 disease showed a radical resection rate of 98.4% [18].

Herb published the largest retrospective study published on this issue which evaluated the trends and outcomes of MIS for locally advanced (IIIA-N2) NSCLC compared to open surgery. A radical resection rate of 94.1% was found in 459 robotic lobectomies [27]. A randomized trial by Huang, comparing RATS (*N* = 58) with thoracotomy (*N* = 55) in clinical N2 patients, showed a radical resection rate of 98.2% [28]. Park evaluated of 31 MIS for locally advanced disease (17 Robotic and 14 VATS) and reported a radical resection rate of 97% in the MIS group as a whole [20].

### Conversion to thoracotomy, learning curve and independent risk factors

In ths report, a conversion to thoracotomy rate of 22.1% was found. Previous studies report rates of 2.8–26%. A conversion to thoracotomy rate of 2.8% was described in lobectomies for cTNM IIB–IIIA NSCLC [17]. In three studies in 210, 459 and 113 patients with clinical or occult mediastinal disease, RATS was converted in 9.9, 9.0 and 8.6% of the procedures, respectively [18, 27, 28]. In the smallest cohort of 31 MIS procedures (17 RATS), a conversion rate of 26% was seen [20].

The high incidence of T3–T4 tumors (68.4%) with a central tumor location and association with ingrowth in vascular structures possibly explains the need for pneumonectomy (*n* = 17) and relation to conversion. A significant increase in the overall proportion of open lobectomies when T stage increased was described previously by Herb [27].

Pneumonectomies were completed by RATS in 47.1% in this cohort. This is lower than the rates in two recent studies comparing cohorts of minimally invasive pneumonectomy to an open approach, reporting 61.5 and 59.4% completion of pneumonectomy by RATS by Gao [29] and Hennon [30]. No doubt that the surgeon’s learning curve influences the conversion rate. This is illustrated by a decline in converted pneumonectomies during the course of the study, from 21% in 2015 to 12.5% in 2017 and none in 2020. In line with this, 80% of the irradical resections occurred in the first 2 years.

Independent risk factors for conversion were pneumonectomy, squamous cell histology, induction therapy and additional resection. Recently, a study by Chen [31] found lobectomy and tumor size < 5 cm as a protective risk factors for conversion in MIS (RATS and VATS) compared to other resections, including pneumonectomy. This is in line with our study in which we found left upper lobectomy to be significantly associated with conversion in univariate analysis, but it was no longer predictive in the final multivariate model. This contrasts with the finding of higher conversion rates and learning curve for right upper lobe resection (and pleural adhesions) in a previous report [32]. However, it seems that upper lobe resections and higher conversion rates are associated and probably require prolonged learning curves [32]. In this series, a few patients had induction treatment and this was identified as an independent risk factor for conversion. This confirms a previous finding in pretreated clinical N1–N2 disease operated with RATS [33].

### Local recurrence

In this cohort, a 2-year recurrence-free survival of 53.8% was found which is comparable with the 3-year recurrence-free survival in three previous studies varying from 37.7 to 49.0% [17, 18, 20].

All studies estimated recurrence-free survival results in cohorts selected on mediastinal involvement and not particularly on T stage, which might affect prognosis negatively.

Regarding pneumonectomies, Gao [29] and Hennon [30], both compared a minimally invasive approach with open pneumonectomy. Gao found no significant differences in recurrence-free survival (40.3 vs. 42.9%) and overall survival, also after propensity score matching. Hennon found improved overall median survival for MIS pneumonectomy.

### Complications

The major complication rate of 10.5% (according to Clavien–Dindo grade III–V) as observed in this analysis was equal to the rate in a larger retrospective study by Veronesi [18].

Overall, 44 complications occured in 34 patients (35.8%), which is higher compared to the overall complications rates of 27.6% and 13.9% found by Huang [28] and Li [17] respectively. Thirty-two of the 44 complications were grade I–II Clavien–Dindo complications and could be treated either conservatively or pharmacologically. Noteworthy is that the Clavien–Dindo classification is not used by all investigators which might have an effect on the interpretation of the results. The largest retrospective cohort analysis did not report on complications, but showed a lower 90-day mortality odds compared to VATS (OR 0.42) [27].

### Strengths

To our knowledge, this is one of few sizeable studies conducted in patients with advanced stages of NSCLC after exclusion of unforeseen N2 disease by RATS, in which the emphasis lies on the technical feasibility instead of on the lymph node status. Workup of all patients was performed by a multidisciplinary team according to the latest guidelines and all surgical resections were included and performed by a single surgeon. By using standardized definitions for recurrence and complication rates, we were able to compare our results with recent literature. We were able to depict a general overview of the potential of RATS, including various anatomical resections. This potential is underlined by Geraci [34] who describes a series of complex robotic procedures including pneumonectomy, sleeve lobectomy, left upper lobectomy and tumor masses larger than 5 cm with or without mediastinal involvement. The role of surgery in higher stages of NSCLC will increase because of the promising results of induction with chemotherapy and immune therapy combinations [35].

### Limitations

First, the single-center, single-surgeon nature of this study makes it prone to bias. By including both clinical and pathological stages of NSCLC (IIB–IVA), a degree of upstaging or downstaging was present in this study. Patients with stage IIA NSCLC were intentionally not included, while they could have added to the power of the study.

Second, comparison of RATS to VATS was not feasible, as RATS was the predominant minimally invasive surgical technique for resections of NSCLC in our center.

Third, due to the retrospective origin of the study, there were missing data in the follow-up and therefore the effect of neoadjuvant therapies on recurrence or survival could not be analyzed.

Furthermore, as time progresses, another limitation of this study is that the robotic platform that was used has now deprecated. The platforms that are currently in use are the Da Vinci X and Xi which are technologically more advanced in comparison to the Si system. Finally, it is hard to compare results with other cohorts reported in literature, since patient cohorts differ enormously in stage and performed procedures. For instance, the advanced tumor stage in the previously mentioned studies are primarily based on lymph node-associated N2 disease and consist of lobectomy procedures mainly.

## Conclusion

In conclusion it is feasible to approach higher-staged NSCLC tumors (especially higher T stages and pretreated patients) with RATS. RATS has a high radicality rate with reasonable conversion to thoracotomy and complication rates that is obviously influenced by the surgeon’s learning curve and finally results in an acceptable local recurrence rate.

## Definitions

### Centrally localized tumor

An endoscopically visible tumor in the central respiratory tract, lobar bronchus or segmental bronchus or otherwise located in direct contact with bronchiole, or vascular structures on CT scan.

### Local recurrence

Tumor recurrence found in the ipsilateral lung, including ipsilateral lymph nodes compared to primary tumor location.

### Distant metastasis

Cancer that has spread from the original (primary) tumor to distant organs or distant lymph nodes.

### Vascular invasion

Invasion of the central vessels, such as the left or right main pulmonary artery, or left or right pulmonary veins.

## Data Availability

All data are available in the Dutch Lung Cancer Audit (DLCAS 2019, Version 1.0 3).
